# Filipino Immigrants in Santa Cruz de Tenerife, Spain: Health and Access to Services

**DOI:** 10.3390/healthcare12131317

**Published:** 2024-07-01

**Authors:** Melynn Grace Parcon, Sara Darias-Curvo, Cristo Manuel Marrero-González, Ángel Ramón Sabando-García

**Affiliations:** 1 Program in Medical and Pharmaceutical Sciences, Development and Quality of Life, University of La Laguna, 38200 Santa Cruz de Tenerife, Spain; 2 Centre for the Study of Social Inequality and Governance, University of La Laguna, 38200 Santa Cruz de Tenerife, Spain; 3 Faculty of Health Sciences, Nursing Section, Department of Nursing, University of La Laguna, 38200 Santa Cruz de Tenerife, Spain; 4 Department of Statistics, Pontifical Catholic University of Ecuador, Santo Domingo 230203, Ecuador

**Keywords:** healthcare, migration, immigrant, insurance, Filipina, Spain, Philippines

## Abstract

The Philippines is a source of labor for many countries. Roughly 10 million overseas Filipinos are working and living outside of the Philippines. This paper examines the association between sociodemographic characteristics (sex, age, educational level, and income) and self-rated physical and mental health, access to healthcare, and health habits among immigrant Filipinos living in Santa Cruz de Tenerife, Spain. Through convenience sampling, Filipino migrants (n = 103) aged 18 years and above participated in the online survey between October 2022 and March 2023. The data were analyzed using descriptive statistical analysis and chi-square. Almost all respondents self-rated their health as excellent and very good. Female respondents are more affected by mental health. Most are enrolled in the Universal Health System of Spain (public insurance). There is more utilization of private health insurance among respondents aged 60 years and above and high-wage earners. Cigarette smoking and alcohol drinking are associated with males. More than half of the respondents perform weekly exercise occasionally or never. These findings suggest a potential need for targeted interventions with an emphasis on the practice of preventive health and the promotion of healthy lifestyles, especially among financially disadvantaged migrants with lesser health access.

## 1. Introduction

The global estimate of international migrants in the world was 281 million in the year 2020 [1]. The pursuit for a higher quality of life and improvement of the socio-economic position of the migrant and their family serve as the main reasons for migration [2].

The Philippines is a source of labor for many countries. Roughly 10 million overseas Filipinos are working and living outside of the Philippines. In 2021, there were a total of 1.83 million overseas Filipino workers (OFWs). Women comprise 59.6% of the total workers, while 40.4% are men. Most of the workers belong to the age group of 30–39 years old. The majority of OFWs are engaged in manual labor [3]. 

### 1.1. Contribution of Migrants

From April to September 2021, the remittances sent by Filipino workers abroad reached PHP 151.33 billion (USD 2.7 billion), which accounted for 9% of the gross domestic product of the Philippines in the year 2020 when the COVID-19 pandemic was at its peak. Even during a global recession, OFWs sent money to their families in the Philippines and, consequently, helped the Philippine economy stay afloat during this difficult time [4]. In their destination country, racialized migrants have also contributed to the economy during the COVID-19 pandemic. For instance, those working in the food sector and caregivers have contributed to the provision of the basic needs of the community, oftentimes putting their health at risk [5].

### 1.2. Barriers and Difficulties for Filipino Migrants to Access Healthcare Services

Migrants experience barriers to health access in their destination country, significantly impacting their health outcomes. Among these barriers are legal restrictions, documented elsewhere to limit healthcare access for specific populations (e.g., undocumented immigrants). Furthermore, language and cultural disparities were observed to hinder effective communication and potentially erode trust in the medical system. Financial constraints, likely due to a lack of health insurance or limited income, may compound these challenges, potentially restricting access to necessary healthcare services [6,7].

A study in the U.S. affirms that immigrants have lower rates of health insurance, use less healthcare, and receive lower quality of care than U.S.-born populations [8]. As cultural beliefs and practices influence the health-seeking behavior of migrants [2,9], the country of origin is an important determinant of health. Culturally appropriate health-promotion programs are necessary for migrants considering diversity, cultural identity, socio-economic status, and education. There is a need to study the specific health needs and health situations of each migrant group [2,9,10].

### 1.3. Consequences of Marginalization 

The situation of language barriers, poor adaptation to the new country, the absence of familial support, a lack of legal status, and the poor enforcement of labor protections in the adopted country add to the difficulty of accessing proper healthcare and open the door to violence, marginalization, exclusion, discrimination, and exploitation [5,11,12,13,14]. In addition, migrants often work for less pay, for longer hours, and in worse conditions than non-migrants, and engage in jobs that are hazardous to health. In Barcelona, Spain, many Filipinos reportedly takes illegal drugs to be able to work for 72 h [15]. This leads to poor health outcomes, workplace injuries, and occupational death [16].

During the COVID-19 pandemic, it was impossible to follow public health protocols such as social distancing, self-isolation, quarantining, or handwashing due to housing conditions. Emergency departments are inaccessible for non-status migrants [17]. In the U.K., when remote services were implemented during the pandemic, consultations for vulnerable migrants dropped to under half the pre-pandemic numbers [18]. The COVID-19 pandemic has increased the vulnerability and marginalization of this group.

On the other hand, social, familial, emotional, and informational support leads to better access to healthcare among migrant Filipinos [19,20,21].

### 1.4. Justification and Purpose of This Study

The United Nations Educational, Scientific and Cultural Organization (UNESCO) [22] states the following:

In applying and advancing scientific knowledge, medical practice, and associated technologies, human vulnerability should be taken into account. Individuals and groups of special vulnerability should be protected, and the personal integrity of such individuals respected.

It is the right of every person regardless of societal status to receive adequate and quality healthcare services, most especially the migrant population, who are most likely prone to being marginalized due to their lack of knowledge of the language of the host country, the lack of recognition of their educational attainment, and dangerous living conditions. As a consequence, this situation makes them more susceptible to developing health problems [23]. 

Moreover, it is of the essence to include their health needs in national plans, policies, and strategies as the effect of migration poses an important public health issue, not only for the next generation; hence, it must be tackled at once [24].

Various studies exist regarding the health of Filipino migrants in countries such as the U.S., Canada, Israel, China, etc., but no research exists regarding the migrant Filipino community in Spain, even though 200,000 Filipinos were living in this country in 2022 [25].

The findings of this study will provide a valuable reference for policymakers and healthcare providers that seek to uplift the quality of healthcare that this population receives through the careful and methodical analysis of the familial situation, gender, socio-economic, and cultural milieu. According to WHO, improving the conditions of daily life is essential for the rapid reduction in social inequalities in health [26]. 

Therefore, the objective of this study is to examine the association between sociodemographic characteristics (sex, age, educational level, and income) and self-rated physical and mental health, access to healthcare, and health habits among immigrant Filipinos living in Santa Cruz de Tenerife, Spain.

## 2. Materials and Methods

### 2.1. Setting

The study was carried out in Santa Cruz de Tenerife, the Canary Islands, Spain, between October 2022 and March 2023.

### 2.2. Study Design 

This is a cross-sectional descriptive study using a newly created self-administered questionnaire directed at immigrant Filipinos.

### 2.3. Participants

The study included Filipino migrants from 18 to 80 years old living in Tenerife, the Canary Islands. We used convenience sampling to capture as many respondents as possible. 

One of the authors, who is Filipino, contacted the leaders of the Filipino communities through the Philippine Consulate of the Canary Islands. Invitation to answer the questionnaire was posted on the social media (Facebook page) of the Philippine Consulate of the Canary Islands as well as the respective social media chat groups of Filipino Community Organizations. Invitations to participate in the study were also given through face-to-face invitations.

### 2.4. Variables

The independent variables included in this study are the sociodemographic profiles of the respondents. which include sex, age, educational level, and monthly income. The dependent variables are health access and health condition, health habits and lifestyle, and mental health. Other sociodemographic data that were included in the survey but not in the statistical analysis are religion, civil status, duration of stay in Spain, place of birth, migration status, type of job, and the ability to speak and understand the Spanish language. 

### 2.5. Data Sources

The questionnaire was constructed by the researchers after a literature review. It was then validated by 5 professors from the Universidad de La Laguna, 1 professor from the Pontifical University of Ecuador, and 1 clinical psychologist from Honduras.

Afterward, pilot testing was conducted with 10 Filipino migrants. The purpose of the pilot study was to evaluate the instrument in terms of the feasibility, appropriateness, and clarity of the questions, which enabled the researchers to improve the questionnaire. The final questionnaire contained 48 questions of multiple-choice type. Both the pilot test questionnaire and the final questionnaire are written in both Filipino (Tagalog) and English languages. The survey and research information were available online through Google Forms. 

Recruitment was carried out after church services, during social gatherings at places of worship, and at the Filipino community events center where formal meetings and informal gatherings are held such as the celebration of festivities, birthdays, and anniversaries. The organization leader introduced the Filipino researcher to the community, explaining the presence of the latter. In turn, the Filipino researcher communicated to the group the objective of the research, the voluntary and anonymous nature of the study, as well as the option to not participate in the study if they did not wish to do so. The aim of the study was written in the survey and explained verbally, and questions from participants were answered before they responded to the survey. Participants were also recruited by approaching business establishments such as nail salons and restaurants where Filipinos were working. Several participants were also recruited through the Facebook page of the Consulate of the Philippines in the Canary Islands as the survey link was also posted there. The manner of answering the survey was online and face to face. Assistance in responding to the survey was given to those who had difficulty with technology. Consent was assumed when the participants agreed to answer the survey. 

### 2.6. Study Size

A total of 103 subjects aged 18 and above were included in the study. As of 2023, the number of Filipinos living in Santa Cruz de Tenerife, Spain, is 466 (all ages) [27]. 

### 2.7. Statistical Methods

After data collection, analysis of the data was carried out through IBM SPSS STATISTICS 25. The following statistical analyses were carried out: descriptive statistical analysis and chi-square to establish the association between the independent and dependent variables. All variables were categorical. Significant association was set at *p* > 0.005.

### 2.8. Ethics Statement

The study was reviewed and approved by the Ethics Committee of the Universidad de La Laguna with code CEIBA 2023-3281. The confidentiality of the data collected is protected by Spanish legislation of Organic Law 3/2018, of December 5, on personal data protection and the guarantee of digital rights.

## 3. Results

The total number of participants in the survey was 105. A total of 103 participants were included in the final analysis after eliminating participants with incomplete data.

### 3.1. Sociodemographic Profile of the Study Population

Almost 45% of respondents belong to the age range of 43–59 years (n = 46), the majority identified themselves as female (73.8%), while 78% belong to the Roman Catholic faith. More than half of the respondents (62.1%) are married or in a common-law partnership. In terms of education, 53.4 % have college and postgraduate levels. On the other hand, 46.6% have high-school-level education or lower. Nearly 50% of the respondents have been living in Spain for 5 to 20 years, while 45.6% have been living in this country for more than 20 years. Almost all the respondents were born in the Philippines (94.2%) and 68.9% are Spanish (EU) citizens. Among all the respondents, 70.9% perform manual types of labor, while less than half of the respondents (46.6%) earn between EUR 1001 and 1800 monthly. Close to half of the population rated themselves as having the ability to speak (46.6%) and understand (43.7%) the Spanish language quite well (Table 1).

### 3.2. Health Access and Health Condition

In the study sample, 75% reported knowing the available health services in their community, while 71% rated the health services received as above average and excellent. With regard to health insurance access, 98% of the respondents reported that it is affordable for them, and almost 80% are enrolled in the Universal Health System of Spain. However, 41.7% of the population expressed that they undergo routine check-ups only when the need arises; therefore, preventive health measures being offered, such as screening tests for the early detection of illness, are not being utilized to the fullest. On the other hand, 45% of the respondents have hypertension and/or hypercholesterolemia and 79% claimed that their medical condition was diagnosed by a medical doctor. It is significant to point out that almost 60% of the respondents have used traditional Filipino healing practices while in Spain (Table 2).

### 3.3. Health Habits and Lifestyle

The health habits and lifestyle of the population were also examined, with 97% of the study participants indicating that they do not smoke, while 47% consume alcohol.

Among those who consume alcohol, 36% indicated that they consume it with a frequency of once a month or less. In terms of physical activity, 42.7% of the population reportedly exercise occasionally or at least once a week, while 14% do not exercise at all. Additionally, almost 60% described their quality of sleep as normal, and 71.8% have an average of 6 to 9 h of sleep.

Notably, only 5% of the population rated their health as ’’bad’’, although 45% of the respondents reported having hypertension and/or hypercholesterolemia (Table 3).

### 3.4. Mental Health

Regarding the mental health of the study participants, 31.1% have experienced difficulties with work or daily life due to emotional stressors sometimes, while 48.5% revealed that their mental health has not affected their ability to finish their work. Likewise, 60.2% had not felt dejected for more than 2 weeks in a row, and 53.4% of the respondents stated that their relationships were not affected by emotional disturbances in the past two weeks (Table 4).

### 3.5. Bivariate Analysis

Bivariate analysis (chi-square) was conducted to determine if an association exists between the sociodemographic profile and the access to health services, health habits and lifestyles, and mental health of the subjects (Table 5). The results presented have a *p*-value of less than 0.005.

Cigarette smoking and alcohol drinking are associated with Filipino males. 

Most of the respondents belonging to the age group 18 to 42 years old (65.7%) and 43 to 59 years old (63.1%) exercise occasionally or none every week. Likewise, most respondents whose monthly salary income is EUR 800 to 1000 (63.1%) and EUR 1001 to 1800 (68.8%) exercise occasionally or not at all every week.

The female respondents are more affected by mental health issues, as 14% reported that their mental health “somewhat often” impacts their ability to work, compared to only 1% of males. 

The study population belonging to the group aged 60 years old and above uses more private insurance (36.4%) than younger age groups. As monthly income augments, the use of private health insurance also increases. In the group with the highest monthly income (EUR 1800 and above), 43.8% use private health insurance. In contrast, the group with the lowest monthly income (less than EUR 800) has the highest number of uninsured individuals (20%).

## 4. Discussion

This study is the first to assess the perceived health, access to healthcare, and health habits of immigrant Filipinos in Spain. These findings indicate that some health deficits are unique to Filipino immigrants such as a lack of knowledge about health and diseases and paucity in the practice of healthy habits. 

Although 98% of the study sample rated their health as excellent and very good, a concerningly high prevalence of chronic disorders was identified among Filipino migrants in Tenerife, as almost half (45%) self-reported having hypertension and/or hypercholesterolemia. The discrepancy between self-assessed health and objective disease burden suggests a significant health literacy deficit in this population. This gap may indicate that individuals do not have an adequate understanding of their health conditions, which could be the result of educational, cultural, or health information access barriers. In addition, a lack of knowledge about disease prevention and management could contribute to an underestimation of their severity and a delay in seeking appropriate medical care. This knowledge deficit is critical, as it may lead to poorer health prognosis and increase the long-term burden of disease, emphasizing the need for educational and public health interventions aimed at improving health literacy in this population. People with poor health literacy may use less preventive healthcare services [28].

In congruence with our data, research carried out with Filipino migrants in the U.S. and Australia found that there is a high prevalence of cardiometabolic disorders, dyslipidemia, hypertension, diabetes, metabolic syndrome, hyperuricemia, and gout in comparison with other races. Despite the presence of these conditions, Filipinos rate themselves as having good and excellent health [2,8,20].

Several factors may contribute to this disparity. A 2018 national survey in the Philippines identified potentially detrimental health behaviors among Filipinos in general, including the low consumption of fruits, vegetables, and whole grains; physical inactivity; binge drinking; and smoking [20,29]. In this study, alcohol consumption (66.7%, n = 18) and cigarette smoking (14.8%, n = 4) are associated with males (Table 5). 

Filipino migrant women face significant mental health challenges, as evidenced by a gender disparity in work productivity. Notably, 14% of female respondents reported that their mental health “somewhat often” impacts their ability to work, compared to only 1% of males (Table 5). This finding aligns with existing research highlighting the vulnerability of Filipino migrant women to mental health issues [30,31].

There were multiple reasons why this difference arose. Physical separation from family, the pressure to elevate the family’s socio-economic status through working long hours, an increase in workload [30,32], and complex Filipino family dynamics can all be significant stressors. While economic empowerment may provide a sense of agency for women, it can also lead to resentment towards partners who fulfill traditionally female domestic roles, particularly during periods of difficulty abroad. 

While a significant portion of Filipino migrants in Tenerife (53.4%) reported having college-level or postgraduate qualifications (Table 1), their employment opportunities reveal a stark contrast. A majority (70.9%) perform a manual type of labor, primarily as domestic helpers or caregivers for women and restaurant jobs such as cooks or waiters for men (Table 1). While some entrepreneurship exists (restaurants and nail salons), upward career mobility is limited. This discrepancy between educational attainment and career trajectory aligns with findings by Lightman et al. [33] regarding Filipina caregivers in Canada. Their study suggests that gender and national origin can act as barriers to upward mobility, with Filipina migrants earning significantly less than other nationalities. This disparity may be attributed to the “racialized Filipina identity” associated with excellence in low-wage care jobs while lacking the perceived competence for higher-paying, complex roles compared to other nationalities [33]. Also, in non-English-speaking countries like Spain, a preferential hiring bias towards Filipinas for domestic and caregiving roles compared to other nationalities appears to exist because of their educational level and knowledge of the English language, which is advantageous for families with children learning English [34].

Our data reveal that more than half of the respondents (58%) reported performing occasional exercise or none every week. This is mostly seen among younger respondents in the age brackets of 18 to 42 years old (65.7%) and 43 to 59 years old (63.1%) in comparison with those aged 60 years old and above (27.3%). Similarly, the same result is observed among respondents with monthly income of EUR 800 to 1000 (63.1%) and EUR 1001 to 1800 (68.8%) (Table 5). Younger individuals and those with low-income levels seem to be more likely not to engage in regular exercise.

Despite seemingly favorable healthcare accessibility indicators, Filipino migrants in Spain exhibit concerning underutilization of preventive health services. While 98% of the study sample reported affordable health insurance, 72.8% are enrolled in the Universal Health System of Spain (Sistema Nacional de Salud), and 75% claimed knowledge of available services (Table 2), a significant portion (41.7%) only seek medical attention upon experiencing symptoms (Table 2). This reactive approach to healthcare suggests missed opportunities for preventative care and early intervention.

This finding aligns with research on healthcare utilization among immigrant populations in other contexts. Similar delayed healthcare seeking among African migrants in the U.S. is present, with doctor visits postponed until illness becomes severe or significantly impacts daily life [35]. Likewise, immigrants’ healthcare utilization is often limited by legal restrictions, excessive bureaucracy within the healthcare system, and negative attitudes from healthcare workers [36,37]. 

More participants belonging to the age group 60 years and above (36.4 %) use private health insurance in comparison to the age groups of 18 to 42 years (17.1%) and 43 to 59 years (19.6%). Likewise, immigrants within the highest income group have more access to healthcare services, as evidenced by the increased use of private health insurance by respondents with a monthly income of more than EUR 1800 (43.8%) in comparison with those with a monthly income of less than EUR 800 (20 %), EUR 800 to 1000 (5.3%), and EUR 1001 to 1800 (22.9%). 

On the other hand, respondents with a monthly income of less than EUR 800 reported that they have no health insurance (20%), in contrast with respondents whose monthly income is EUR 800 to 1000 (0%) and more than EUR 1800 (2.1%) and EUR 1001 to 1800 (0%). Being financially disadvantaged and belonging to a minority group are parallel with findings associated with poor health outcomes [28]; hence, actions should be undertaken to bridge the gap of access to healthcare amongst immigrants.

## 5. Conclusions

The health needs of immigrant Filipinos in Santa Cruz de Tenerife, Spain, suggest a potential need for targeted interventions with an emphasis on health concerns unique to Filipino immigrants such as improving overall health knowledge and habits, especially among younger individuals. The prevention and control of dyslipidemia and hypertension, as well as providing mental healthcare, especially for women, are also needed. Financially disadvantaged immigrants have less access to insurance; hence, actions must be oriented toward the provision of healthcare to this marginalized population. Healthcare personnel working with immigrant communities require a deeper understanding of cultural perspectives on health and illness across different migrant groups as the health needs of every population are unique; hence, there is a need for healthcare professionals trained in public health, social inequalities, cultural competence, and immigrant health to adequately address the health needs of immigrant populations. 

## Figures and Tables

**Table 1:** Sociodemographic profile of the study population (n = 103).

Variables		n	%
Gender			
	Female	76	73.8
	Male	27	26.2
Age (Years)			
	18–42	35	34
	43–59	46	44.7
	60 and above	22	21.3
Religion			
	Roman Catholic	80	77.7
	Others	23	22.3
Civil Status			
	Single	27	26.2
	Married and common-law partnership	64	62.1
	Separated or widow	12	11.7
Educational Level			
	College or postgraduate	55	53.4
	High school or lower	48	46.6
Duration of Stay in Spain			
	Less than 5 years	5	4.9
	5–20 years	51	49.5
	More than 20 years	47	45.6
Place of Birth			
	Philippines	97	94.2
	Spain	6	5.8
Migration Status	Irregular	1	1
	Spanish citizen	71	68.9
	Foreign worker	31	30.1
Type of Job			
	Manual	73	70.9
	Non-manual	4	3.9
	Not working	26	25.2
Monthly Income (EUR)			
	Less than 800	20	19.4
	800–1000	19	18.4
	1001–1800	48	46.6
	More than 1800	16	15.5
Can Speak Spanish			
	A little	32	31.1
	Quite well	48	46.6
	Very well	23	22.3
Can Understand Spanish			
	A little	28	27.2
	Quite well	45	43.7
	Very well	30	29.1

**Table 2:** Health access and condition of the study population (n = 103).

Variables		n	%
Do you know the available health services in your community?			
	Yes	75	72.8
	No	11	10.7
	I only know some health services	17	16.5
How do you rate the health services in your area?			
	I don’t know	3	2.9
	Below average	3	2.9
	Average	26	25.2
	Above average/Excellent	71	68.9
Is health insurance affordable for me?			
	Yes	98	95.1
	No	5	4.9
If yes, please specify the type of insurance that you have			
	Private	23	22.3
	Public	75	72.8
	Not applicable—I don’t have health insurance	5	4.9
Frequency medical check-up			
	Only when needed	43	41.7
	Once in 3 to 6 months	36	35
	Once a year	22	21.4
	I don’t go to medical check-ups	2	1.9
Medical condition diagnosed by a doctor			
	Yes	79	76.7
	I don’t have medical problems	24	23.3
Migrant with hypertension and hypercholesterolemia		45	41.7

**Table 3:** Health habits and lifestyle of the study population (n = 103).

Variables		n	%
Cigarette smoking			
	I don’t smoke	97	94.2
	10 or fewer	6	5.8
Alcohol consumption			
	No, never	56	54.4
	Yes	47	45.6
Frequency of alcohol consumption			
	4 or more times per week	1	1
	2–3 times per week	3	2.9
	2–4 times a month	7	6.8
	Monthly or less	36	35
Frequency of exercise			
	I don’t exercise	14	13.6
	I exercise occasionally at least once a week	44	42.7
	I exercise 3 to 5 times a week	26	25.2
	I exercise every day	19	18.4
Quality of sleep			
	Very Good	11	10.7
	Good	14	13.6
	Normal	61	59.2
	Bad	17	16.5
Number of hours of sleep per day			
	Less than 4 h	3	2.9
	4 to 6 h	25	24.3
	6 to 9 h	74	71.8
	No response	1	1
Self-rated health quality			
	Bad	5	4.9
	Good	57	55.3
	Excellent	41	39.8

**Table 4:** Mental health of the study population (n = 103).

Variables		n	%
Have you had any problems with your work or daily life due to any emotional problems, such as feeling depressed, sad, or anxious?			
	Yes	8	7.8
	No	63	61.2
	Sometimes	32	31.1
How often has your mental health affected your ability to get work done? For example, unable to concentrate while working			
	Somewhat often	11	10.7
	Not so often	40	38.8
	Not at all	50	48.5
	Very often	2	1.9
Have you felt particularly low or down for more than 2 weeks in a row?			
	Yes	7	6.8
	No	62	60.2
	Sometimes	34	33
During the past two weeks, how often has your mental health negatively affected your relationships?			
	Somewhat often	6	5.8
	Not so often	39	37.9
	Not at all	55	53.4
	Very often	3	2.9

**Table 5:** Bivariate analysis.

	Male	Female			x^2^	*p*
	(n = 27; 26.2%)	(n = 76; 73.8%)				
Cigarette smoking per day						
I don’t smoke	23 (85.2%)	74 (97.4%)			5.39	0.02
10 cigarette sticks or fewer	4 (14.8%)	2 (2.6%)				
Consume alcoholic drinks						
No, never	9 (33.3%)	47 (61.8%)			6.527	0.011
Yes	18 (66.7%)	29 (38.2%)				
	18 to 42 years old	43 to 59 years old	59 years old and above			
	(n = 35; 34.0%)	(n = 46; 44.7%)	(n = 22; 21.3%)			
I don’t exercise	7 (20%)	5 (10.9%)	2 (9.1%)		13.052	0.042
I exercise occasionally at least once a week	16 (45.7%)	24 (52.2%)	4 (18.2%)			
I exercise at least 3 to 5 times a week	9 (25.7%)	8 (17.4%)	9 (40.9%)			
I exercise every day	3 (8.6%)	9 (19.6%)	7 (31.8%)			
Monthly Income						
	less than EUR 800	EUR 800 to 1000	EUR 1001 to 1800	more than EUR 1800		
	(n = 20; 19.4%)	n = 19 (18.4%)	(n = 48; 46.6%)	(n = 16; 15.5%)		
I don’t exercise	3 (15%)	7 (36.8%)	2 (4.2%)	2 (12.5%)	28.377	0.001
I exercise occasionally at least once a week	6 (30%)	5 (26.3%)	31 (64.6%)	2 (12.5%)		
I exercise at least 3 to 5 times a week	6 (30%)	4 (21.1%)	8 (16.7%)	8 (50%)		
I exercise every day	5 (25%)	3 (15.8%)	31 (64.6%)	2 (12.5%)		
	Male	Female				
Mental health affects the ability to finish work	(n = 27; 26.2%)	(n = 76; 73.8%)				
Somewhat often	1 (3.7%)	10 (14%)			7.539	0.057
Not so often	7 (25.9%)	33 (45%)				
Not at all	19 (70.4%)	31 (42%)				
	18 to 42 years old	43 to 59 years old	59 years old and above			
Type of Insurance	(n = 35; 34.0%)	(n = 46; 44.7%)	(n = 22; 21.3%)			
Private	6 (17.1%)	9 (19.6%)	8 (36.4%)		8.991	0.061
Public	28 (80%)	36 (78.3%)	11 (50 %)			
None	1 (2.1%)	1 (2.2%)	3 (13.6%)			
Monthly Income						
	less than EUR 800	EUR 800 to 1000	EUR 1001 to 1800	more than EUR 1800		
Type of Insurance	(n = 20; 19.4%)	n = 19 (18.4%)	(n = 48; 46.6%)	(n = 16; 15.5%)	20.071	0.003
Private	4 (20%)	1 (5.3%)	11 (22.9%)	7 (43.8%)		
Public	12 (60%)	18 (94.7%)	36 (75%)	9 (56.3%)		
None	4 (20%)	0 (0%)	1 (2.1%)	0 (0%)		

## Data Availability

The data presented in this study are available from the corresponding author upon request.
